# Thoracic lymphangitis as an immune-related adverse event: a case report

**DOI:** 10.1186/s12890-024-03123-5

**Published:** 2024-06-25

**Authors:** Haruki Kobayashi

**Affiliations:** Division of Thoracic Oncology, Shizuoka Cancer Center, 1007 Shimonagakubo, Shizuoka, Nagaizumi-Cho Sunto-Gun 411-8777 Japan

**Keywords:** Immune-related adverse event, Thoracic lymphangitis, Non-small cell lung cancer

## Abstract

The efficacy of immune checkpoint inhibitors (ICIs) has been widely recognized in several cancers and is now being used in the perioperative setting for lung cancer.

We recently encountered an immune-related adverse event that has not been previously reported: thoracic lymphangitis, which occurred after postoperative ICI treatment for lung cancer. The patient complained of breathlessness and her condition rapidly progressed to hypoxia grade 3. Chest computed tomography revealed significant lymphostasis. With high-dose steroid treatment, the patient showed improvement.

Therefore, as the frequency of neoadjuvant, adjuvant, and perioperative ICI use is expected to increase, it is crucial to understand and monitor this adverse event.

## Background

The efficacy of immune checkpoint inhibitors (ICIs) has been widely recognized for several cancers, and they are now being used in the perioperative setting for lung cancer [1–4], whereas there is a certain proportion of patients who experience adverse events that prevent them from continuing treatment [5, 6]. It is predicted that unexperienced immune-related adverse event (irAE) may occur by administering ICIs in a larger number of cancer patients.

## Objective

We report a novel irAE occurring after postoperative adjuvant chemotherapy for lung cancer.

## Case report

**Fig. 1:**
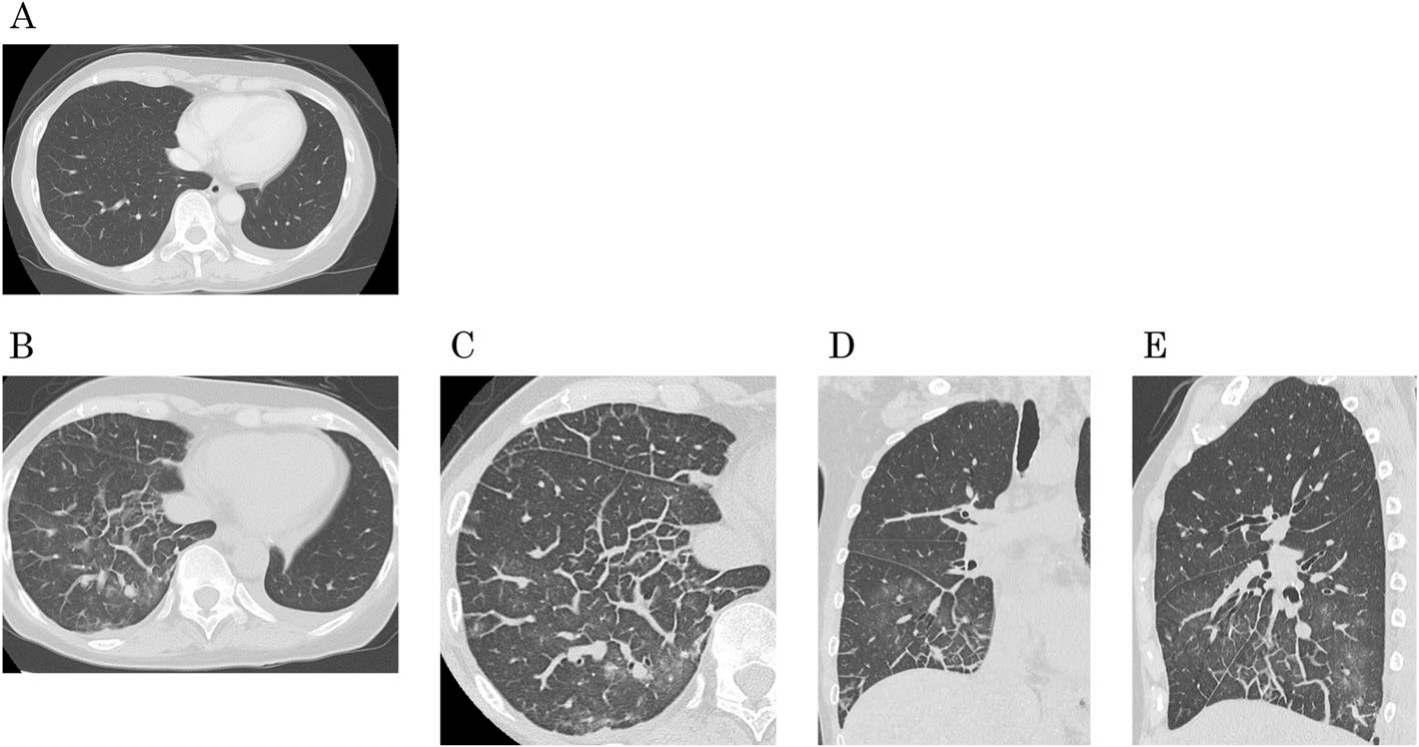
**A** Computed tomography (CT) of chest showed normal post-operative findings. **B** Axial computed tomography (CT) showed severe lymphostasis. **C** Axial, (**D**) Coronal, and (**E**) Sagittal view of high-resolution chest CT showed severe lymphostasis

A 65-year-old woman developed postoperative chylothorax after undergoing left upper lobectomy with wedge bronchoplasty with ND2a-2 for left upper lobe lung cancer. Decreasing dietary fatty acids did not improve her condition; however, lymphangiography with lipiodol closed the leak. Thereafter, the patient was treated for p-stage IIIA non-small cell lung cancer with tumor cell PD-L1 expression 20% (22C3) with four cycles of postoperative adjuvant chemotherapy regimens of cisplatin and vinorelbine on the 59th day postsurgery. Computed tomography (CT) results were normal without recurrence (Fig. 1 A). Based on the IMpower010 trial, atezolizumab was administered. After day 9 of cycle 1, she developed fever in between grade 1 to 2. On day 15, she complained of breathlessness and her condition rapidly progressed to hypoxia grade 3. Chest CT conducted on the same day revealed significant lymphostasis mainly located in right lower lobe (Fig. 1 B–E). Occurrence of lymphangitis carcinomatosa, infectious diseases, and heart failure were ruled out. The patient was diagnosed with lymphangitis, an irAE, due to blockage of the thoracic duct by chylothorax treatment and activation of lymphocyte caused by ICI. Treatment with 1000 mg methylprednisolone was initiated on day 15. The chest X-ray showed complete improvement on day 8 of steroid treatment. The patient could discontinue taking steroids without experiencing recurrence.

On day 15 after atezolizumab treatment, the patient also experienced acute inflammatory demyelinating polyneuropathy as an irAE, which was diagnosed by a neurologist, and intravenous injection of immunoglobulin were administered after steroid treatment. The symptoms of difficulty in walking and limbs dysesthesia gradually improved.

After administering a single dose of atezolizumab, the patient was observed without additional treatment, and the recurrence-free survival time for lung cancer was longer than one year.

## Discussion

We experienced unrecognized irAE, thoracic lymphangitis, which occurred after ICI treatment for lung cancer. The irAE is considered a significant side effect leading to respiratory failure.

The causes for localized lymphangitis in the right lower lobe are considered as follows:

- First, in this case, surgery including bronchoplasty in the left upper lobe was performed, and lymph node dissection was carried out without omission. Therefore, it is believed that lymphangitis did not occur in the left lung.
- Second, the lymphatic pathways in the right upper lobe are unaffected as they drain into the right lymphatic duct through a separate route.
- Third, the lymphatic vessels within the right lower lobe that drain into the inferior tracheobronchial nodes are believed to have developed lymphangitis because of lymphatic congestion resulting from postoperative chylothorax therapy and further exacerbated by the activation of lymphocytes associated with ICI.

No reports of thoracic lymphangitis as an irAE after ICI administration exist. As the frequency of using neoadjuvant, adjuvant, and perioperative ICI is expected to increase in the future, it is crucial to understand and monitor this adverse event.

## Data Availability

No datasets were generated or analysed during the current study.

## Abbreviations

ICIs: Immune checkpoint inhibitors
irAE: Immune-related adverse event
CT: Computed tomography
